# Cognitive-Behavioural Correlates of Dysbiosis: A Review

**DOI:** 10.3390/ijms21144834

**Published:** 2020-07-08

**Authors:** Maria Luca, Siriporn C. Chattipakorn, Sirawit Sriwichaiin, Antonina Luca

**Affiliations:** 1 Department of Medical and Surgical Sciences and Advanced Technologies, “GF Ingrassia”, University of Catania, 95123 Catania, Italy; lucmaria@tiscali.it; 2 Neurophysiology Unit, Cardiac Electrophysiology Research and Training Center, Faculty of Medicine, Chiang Mai University, Chiang Mai 50200, Thailand; scchattipakorn@gmail.com (S.C.C.); sirawit.sriwichaiin@gmail.com (S.S.); 3 Center of Excellence in Cardiac Electrophysiology Research, Chiang Mai University, Chiang Mai 50200, Thailand; 4 Department of Oral Biology and Diagnostic Sciences, Faculty of Dentistry, Chiang Mai University, Chiang Mai 50200, Thailand; 5 Cardiac Electrophysiology Unit, Department of Physiology, Faculty of Medicine, Chiang Mai University, Chiang Mai 50200, Thailand

**Keywords:** microbiota, gut, dysbiosis, behaviour, cognition

## Abstract

Evidence suggests an association between an altered gut microbiota (dysbiosis), cognitive performance and behaviour. This paper provides an overview of the current literature regarding the cognitive-behavioural correlates of dysbiosis, with special attention on the clinical and biochemical mechanisms underlying the association between dysbiosis, cognition (mild cognitive impairment and dementia) and behaviour (depression, schizophrenia, addiction). After providing an overview of the evidence, the review discusses the molecular aspects that could account for the cognitive-behavioural correlates of dysbiosis. Shedding light on this topic could provide insights regarding the pathogenesis of these burdening neuropsychiatric disorders and even suggest future therapeutic strategies.

## 1. Introduction

The gastrointestinal tract (GIT) covers a surface of 250–400 m^2^ and contains over 500 different species of bacteria, constituting the so-called gut microbiota [1,2]. The functions of GIT microbiota are far from being limited to digestion, encompassing the modulation of the immune system, the metabolism of drugs, the synthesis of vitamins and short-chain fatty acids (SCFAs), as well as the inhibition of pathogens [2]. In the light of these fundamental physiological functions, it is apparent that the composition of microbiota influences not only the gastrointestinal homeostasis, but also the general health of the human host [3]. Indeed, a variety of intestinal (i.e., irritable bowel syndrome, coeliac disease) and extra-intestinal (i.e., metabolic syndrome, cardiovascular disorders, rheumatoid arthritis) disorders have been related to dysbiosis [3]. Dysbiosis consists of an altered microbiota, due to the loss of beneficial microbial organisms and microbial diversity, as well as the overgrowth of pathobionts [4]. The latter can be due to several causes, among which the prolonged use of antibiotics, the exposure to psychological stress, as well as an unhealthy diet represent the most studied ones [2]. As far as antibiotics are considered, the broad-spectrum ones can severely damage the intestinal microbiota, potentially resulting in diarrhoea due to the overgrowth of *Clostridium difficile* or the reduced production of SCFAs, contributing to water absorption in the colon [5]. In addition, antibiotics can interfere with the bacterial metabolism of drugs and lead to a decreased resistance to external pathogens, thus exposing the human host to the risk of infections [6,7]. Considering psychological stress, it can cause a significant decrease of Lactobacilli concentration in the faeces, that could be re-conducted to an altered intestinal environment (i.e., reduced gastric acid release and altered motility), induced by stress and threatening Lactobacilli survival [2]. Finally, a diet rich in sulphates can lead to dysbiosis, since the metabolism of sulphates generates toxic by-products, such as hydrogen sulphide, altering the mucosal permeability [8]. Similarly, a diet rich in proteins can cause the production of harmful metabolites, such as indoles, representing co-carcinogens with a potential role in the pathogenesis of bladder and colorectal cancer [2,9]. As previously stated, the consequences of dysbiosis are not limited to the GIT. Indeed, gut microbiota has been demonstrated to affect several functions, some of which may seem to be completely unrelated to the intestinal homeostasis, such as cognition and behaviour [10]. Building on this, our review focuses on the cognitive-behavioural correlates of dysbiosis. For the sake of simplicity, despite being clearly intertwined, cognition and behaviour will be separately discussed in the following paragraphs. After providing an overview of the evidence linking dysbiosis to cognition (mild cognitive impairment and dementia) and behaviour (depression, schizophrenia, addiction), the review then discusses the molecular aspects that could account for the cognitive-behavioural correlates of dysbiosis.

## 2. Cognitive Correlates of Gut Dysbiosis

### 2.1. Gut Dysbiosis and Cognitive Dysfunction: Evidence from Human and Animal Studies

Growing evidence in clinical studies reveals the association between altered gut microbiota and cognitive dysfunction [11,12,13,14,15,16,17]. These studies demonstrated the alterations in five main phyla of gut microbiota including Firmicutes, Bacteroidetes, Proteobacteria, Actinobacteria, and Verrucomicrobia in subjects with cognitive dysfunction [11,12,13,14,15]. In particular, dementia (i.e., Alzheimer’s Disease—AD) has been associated with a reduction in Bacteroidetes and an increase in the Firmicutes/Bacteroidetes or F/B ratio [11,12]. Consistently, a higher level of Firmicutes has been reported in patients with mild cognitive impairment (MCI) [13]. Inconsistently with this evidence, other studies reported a decrease in Firmicutes in AD [14,15]. In a study, the number of Bacteroidetes was found to be increased in amnestic MCI, but no difference was recorded when comparing AD patients and healthy controls [15]. These inconsistent findings might reflect the dynamic changes of gut microbiota during each stage of cognitive dysfunction. Considering the other phyla, the abundance of Proteobacteria and Actinobacteria was shown to be elevated in MCI and AD patients in several studies [12,13,15], while the amount of Verrucomicrobia was shown to be reduced in AD patients [12,13]. An increase in some bacteria belonging to phylum Firmicutes, including Mogibacteriaceae, Phascolarctobacterium, Ruminococcaceae, Enterococcaceae, and Streptococcaceae, have been correlated with cognitive dysfunction [12,13,16]; however, Clostridiaceae, Ruminococcaceae, Eubacteriaceae, Veillonellaceae and Lanchnospiracea are also abundant among people with normal cognitive function [12,15,17]. An increase in Enterobacteriaceae, belonging to Proteobacteria phylum, was shown to be correlated with cognitive impairment in several studies [13,15,17]. Several animal models, including diet-induced obesity (DIO) and transgenic AD model, demonstrated a link between gut dysbiosis and cognitive impairment [18,19,20,21,22,23,24,25,26]. Most of the studies reported that a high-fat diet (HFD) consumption could alter the composition of gut microbiota and lead to further pathophysiological processes in cognitive impairment in HFD-fed animals [18,19,20,21]. The lean mice with normal cognitive function receiving gut microbiota from HFD-fed mice developed cognitive impairment and the reduction in the abundance of *Akkermansia muciniphila,* increased of *Bilophila sp.,* and alteration in the composition of Clostridiales [18]. An increase in the F/B ratio in male Wistar rats treated with HFD for 12 weeks was accompanied by cognitive impairment [19]. A recent study from Saiyasit and colleagues revealed that gut dysbiosis, as indicated by the increased Enterobacteriaceae/Eubacteria ratio, occurred after treating the rats with HFD for two weeks, and resulted in an increased F/B ratio after eight weeks of HFD treatment. Then, HFD-fed rats developed cognitive impairment and brain pathology after 12 weeks of HFD consumption [20]. Deshpande and colleagues also observed an increase in Firmicutes and decreased Bacteroidetes after treated Male Sprague-Dawley rats with HFD; however, the cognitive performance of the treated rats was not different from that characterizing the control group [21]. The strain of animal, duration and composition of the diet may account for the discrepancy in the results. The studies using the transgenic mouse models for AD also revealed a relationship between gut dysbiosis and cognitive impairment [22,23,24,25,26]. Proteobacteria and Verrucomicrobia at the phylum level, Helicobacteraceae and Desulfovibrionaceae at the family level, and *Akkermansia sp.* and *Desulfovibrio sp.* at the genus level were shown to be increased in APP/PS1 mice, while Bacteroidetes at the phylum level and *Lactobacillus sp.* and *Alloprevotella sp.* at the genus level was decreased [22,23]. In the 3xTg-AD mouse model, the F/B ratio was found to be elevated when compared to the control group [24]. Another study found that the abundance of both Firmicutes and Bacteroidetes was increased in 3xTg-AD mice [25]. However, some genus in the phylum of Firmicutes was reported to be lower in 3xTg-AD mice [26]. The variations of results possibly due to different strains of animal, the age of the animal, or analytical techniques. Collectively, all of these animal studies suggested that gut dysbiosis as indicated by an increase in the F/B ratio and increased abundance of Proteobacteria might be associated with cognitive dysfunction. 

### 2.2. Gut Dysbiosis and Cognitive Dysfunction: Possible Underlying Mechanisms

The underlying mechanisms of gut dysbiosis-induced cognitive dysfunction may involve several pathways, including neural, inflammatory and biochemical ones. 

*Neural pathway:* Observations regarding chemical colitis-induced anxiety-like behaviour in the mice demonstrated that the vagus nerve was involved in the pathogenesis of the anxiety-like behaviour and accounted for the beneficial effects of probiotic *Bifidobacterium longum* NCC3001. The role of the vagus nerve seemed to be independent from inflammation and BDNF production [27]. Another study with subclinical infection by *Campylobacter jejuni* demonstrated that the anxiety-like behaviour reported in animals could depend on the direct activation of the neural pathway rather than immune activation [28]. 

*Inflammatory pathway:* Inflammation plays a crucial role in the pathogenesis of cognitive impairment in association with gut dysbiosis. In the time-course study of the DIO model, gut dysbiosis was the initial pathophysiological process to occur after a two-week HFD treatment [20]. Increasing the abundance of gram-negative bacteria could result in a chronic low-grade inflammation in the GIT, impairing the intestinal epithelium and leading to the so-called “leaky gut” [29]. As a consequence, the leakage of pathogen associated molecular pattern, such as lipopolysaccharides, into the circulation could further contribute to systemic inflammation and oxidative stress [19]. The systemic alterations affect the brain, thus favouring the neurodegenerative pathway via the following events: increased neuronal cell apoptosis and brain mitochondrial dysfunction, elevated hippocampal oxidative stress, decreased hippocampal synaptic plasticity, decreased number of dendritic spine density at CA1 area of the hippocampus, microglial over-activation in the hippocampus, increased amyloid-beta deposition [19,20]. The treatments restoring gut microbiota composition are able to attenuate the inflammatory status providing a beneficial effect on cognitive function [19,22,23,25,26,30]. Indeed, several studies demonstrated that the administration of some types of probiotics not only improved the integrity of the intestinal epithelium, but also attenuated the inflammatory status in the GIT [31,32,33]. 

**Figure 1:**
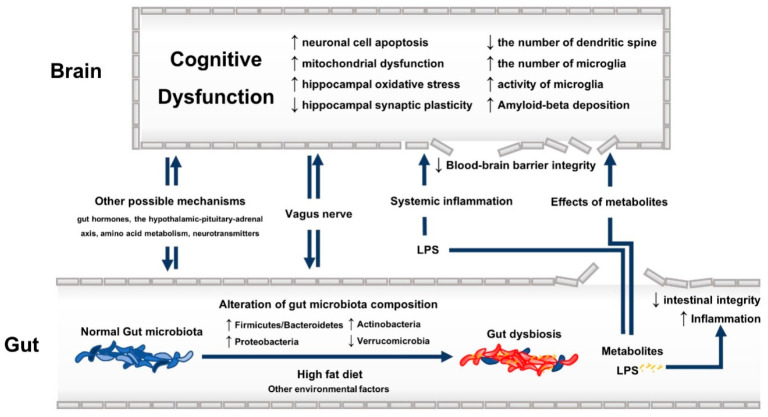
The possible underlying mechanisms of the association between gut dysbiosis and cognitive dysfunction. Up arrow: increased; down arrow: decreased

*Biochemical pathway:* SCFAs, including acetic, butyric, and propionic acid, are one of the potential metabolites of gut microbiota fermentation that can influent cognitive function. Total SCFAs were found to be increased in the faecal content in autism spectrum disorder, the latter being frequently associated with cognitive dysfunction [34]. However, an increase in the level of major SCFAs (acetate, butyrate, and propionate) after treatment with prebiotics has been associated with decreased neuroinflammation in apolipoprotein ε4 allele transgenic mice [30]. Some studies reported the beneficial effects of propionate on the inflammatory process in the intestinal mucosa, which could prevent further systemic inflammation and cognitive dysfunction in mice with colitis [35,36]. In contrast, the level of propionate was found to be associated with cognitive dysfunction when examining the faecal content of mice previously transplanted with gut microbiota from AD patients [37]. Similar results have been reported in mice with cognitive impairment induced by dietary advanced glycation end products [38]. The 3xTg-AD mice also showed elevated propionate levels in both faeces and brain [25]. Animal studies focusing on the effects of acetate and butyrate on cognitive function reported conflicting results [23,30,34,38]. This inconsistency could relate to the different models used to generate cognitive impairment along with the different animal strains. As far as other metabolites are considered, ammonia (a by-product of urease-containing bacteria), has also been linked to cognitive dysfunction [39]. As a matter of fact, it is recognized that the cognitive impairment due to hepatic encephalopathy relates to the accumulation of ammonia (favouring the blood-brain barrier (BBB) breakdown), mainly produced by *Streptococcus salivarius* [39]. Ammonia level was also shown to be elevated in autism spectrum disorder [34]. D-lactate, a metabolite of genera Enterococcus and Streptococcus, has been linked to the cognitive impairment characterizing a sample of patients with chronic fatigue syndrome [16]. Impaired intestinal integrity might be associated with an increase in the serum level of D-lactate [40]. The detrimental effects of D-lactate, such as D-lactic acid encephalopathy, are renown among short-bowel syndrome patients, affected by a bacterial overgrowth leading to considerably high levels of D-lactate [41,42]. Other possible mechanisms that could explain the association between gut dysbiosis and cognitive decline include the modulation of gut hormones [26], the dysfunctional hypothalamic-pituitary-adrenal axis (HPA), the altered amino-acid metabolism and the production of gut-derived neurotransmitters [43,44,45]. See Figure 1 for a visual summary of the possible underlying mechanisms of the association between gut dysbiosis and cognitive dysfunction.

## 3. Behavioural Correlates of Gut Dysbiosis

### 3.1. Gut Dysbiosis and Depression

Major depression is among the most frequent mental disorders. The disease is characterized by a lifetime prevalence of 12% and represents a cause of disability and mortality all over the world [46]. From a clinical point of view, a depressive episode lasts at least two weeks and is characterized by at least five of the following features: depressed mood, anhedonia, apathy, sleep disturbances, changes in appetite, agitation, low energy, cognitive disturbances, thoughts of worthlessness or guilt, thoughts about suicide [47]. 

The pivotal role of dysbiosis in depression has been hypothesized from years. Previous studies have constantly reported the presence of dysbiosis in stressed animals [48]. Similarly, human studies confirmed these observations, demonstrating that depressed individuals present a reduction in microbiome biodiversity, with a relative abundance of Bacteroidetes against a reduction of Lachnospiraceae [49]. Similarly to dementia, altered levels of SCFAs, metabolites of gut microbiota fermentation, can be found in depressed individuals. In particular, a recent study reported that the content of acetic and propionic acid was lower in non-depressed women compared to depressed ones. The latter, on the contrary, showed a higher content of isocaproic acid. Moreover, it should be underlined that the use of some drugs, including lipid-lowering agents, as well as drugs used for dysthyroidism, were found to play a role in SCFAs concentration [50]. Although it has not been completely elucidated, the hypothesis that “depression-associated bacteria” sustain depressed mood (contributing to its pathogenesis through several mechanisms) has been supported by several studies [51].

First of all, it is well known that depressed individuals present an imbalance between anti-inflammatory mediators, such as transforming growth factor-beta and interleukin (IL)-10, and pro-inflammatory ones (i.e., IL-1, IL-6, IL-8, IL-12, tumour necrosis factor-alpha), whereby the latter are over-expressed [52]. During a status of dysbiosis, the intestinal production of high levels of hydrogen sulphide (H_2_S) leads to several detrimental effects in the host, including the decreased mitochondrial oxygen consumption and the overexpression of pro-inflammatory mediator genes, such as that of IL-6 [53]. The hypothesis that dysbiosis sustains a chronic inflammatory status was also supported by the detection, among depressed patients, of high plasmatic concentrations of IgM antibodies against the lipopolysaccharide of Enterobacteriaceae [54].

Moreover, it has been reported that the altered microbiota stimulates the NADPH oxidase and the nitric oxygen (NO) synthesis, thus favouring a pro-oxidative condition and ultimately contributing to the OS-related damage [55].

Furthermore, high concentrations of depression-sustaining hormones, including ACTH and corticosterone, have been detected in stressed germ-free rats [56,57].

Finally, although research has focused primarily on the study of gut bacteria, it should be noted that gut fungal composition has also been associated with depression. Indeed, in depressed individuals, high Candida levels and low Penicillium levels have been reported, thus suggesting the contribution of gut fungal dysbiosis in depression [58].

Building on this, some researchers have conducted pharmacological trials based on the administration of probiotics that could be useful for the treatment of mood disorders. These potential therapeutic tools are sometimes called “psychobiotics”, as to highlight their potential efficacy in reducing the symptoms associated to mood disturbances. However, as a matter of fact there is not enough evidence to claim conclusions on this issue. 

### 3.2. Gut Dysbiosis and Schizophrenia 

Schizophrenia is among the most disabling and economically challenging psychiatric disorders. The WHO ranked schizophrenia in the top ten of disorders strongly contributing to the global burden of disease. The incidence of schizophrenia is 1.5 per 100.000 people with an estimated prevalence of around 1% worldwide. The typical onset is during adolescence, with a slightly higher frequency among men [59]. 

According to the fifth version of the Diagnostic and Statistical Manual for Mental Disorders, the key features for the diagnosis of schizophrenia are: (1) positive symptoms, including hallucinations or delusions and disorganized speech; (2) negative symptoms, including flat affect or poverty of speech, cognitive impairment, alogia, avolition. These symptoms must persist for at least six months and be associated with social or occupational dysfunction [47].

A complex and dynamic interaction between genetic and environmental risk factors has been associated with schizophrenia occurrence. Concerning environmental risk factors, great attention has been paid to prenatal, early post-natal and childhood infections. 

During its development, in fact, the brain is particularly vulnerable to infection; hence, lifelong behavioural and cognitive disorders are likely to occur [60]. Accordingly, several studies have reported an association between maternal Toxoplasma gondii, Chlamydophila pneumoniae, Chlamydophila psittaci and herpes simplex virus type 2 infections with schizophrenia occurrence [61]. 

Although the exact mechanism by which infections increase the risk of schizophrenia occurrence is unclear, a possible explanation could lie in the initiation of an immune response to pathogens, leading to an increased inflammatory state. High levels of circulating pro-inflammatory cytokines are, in fact, frequently observed in schizophrenia and have been associated both to its occurrence and exacerbation [62]. 

Finally, OS-related damage has been found to exert a pathogenic role in schizophrenia, too. Several studies have reported decreased activity of antioxidant enzymes, a reduction of plasmatic antioxidant molecules and an elevated concentration of lipid peroxidation products (i.e., urinary 8-iso-prostaglandin F2α and 4-hydroxynonenal) in subjects with schizophrenia. The detrimental effects of OS should not be neglected, since oxidative damage is a major contributor to neurodegeneration [63,64,65,66].

Interestingly, the reduction of the microbiota variability has been found to be associated with schizophrenia. In particular, compared to healthy subjects, the patients affected by schizophrenia have been found to present an increase of Lactobacillaceae, Halothiobacillaceae, Brucellaceae and Micrococcineae, along with a decrease in the numerosity of Veillonellaceae [67]. These observations are surprising, considering that Lactobacilli are usually the main component of probiotics, believed to be useful for mental health. Moreover, it should be considered that antipsychotics exert an anti-commensal action, thus interfering with microbiota variability [68]. For instance, the antipsychotic olanzapine, known to determine severe weight-gain and diabetes, could account for a decrease in Bacteroidetes and an increase in Firmicutes. [69].

The link between dysbiosis and the pathogenesis of schizophrenia is still debated and further studies are needed in order to shed light on this topic. Notwithstanding, it is known that some dysbiosis-related factors, such as bacterial infections, inflammation and increased BBB permeability, could contribute to the pathogenesis of schizophrenia [70,71]. 

### 3.3. Gut Dysbiosis and Addiction

The perturbations of gut microbiota could significantly contribute to addiction. As mentioned before, the alteration of the gut–brain axis interferes with the HPA stress response, thus favouring addiction and its neuropsychiatric comorbidities, including depression [72]. Moreover, the gut–brain axis could directly influence dopaminergic transmission, closely involved in the reward pathways [73]. Undoubtedly, shedding light on this fascinating topic could lead not only to a better understanding of the mechanisms underlying addiction, but also to the identification of possible therapeutic strategies. 

#### 3.3.1. Alcohol Abuse

According to a recent survey, alcohol use disorder has a prevalence of about 29% and is frequently associated with other substance use disorders as well as other psychiatric disturbances including depression, bipolar disorder, anxiety [74]. Alcohol abuse is often associated with severe medical consequences affecting the whole body, among which gastrointestinal reflux, hepatic failure, electrolyte disturbance, hypertension, atrial fibrillation, dementia, depression [75]. 

A complex interplay of genetic, cognitive, personality and environmental factors seems to play a role in the pathogenesis of alcohol abuse. 

Concerning environmental factors, a potential link between gut microbiota alteration and chronic alcohol intake has been consistently suggested. In particular, several studies reported that individuals with chronically elevated alcohol intake presented an increased small-bowel and colon permeability. As a matter of fact, the toxic effect of ethanol on the small-bowel epithelium has also been reported among healthy individuals and in in vitro studies [76]. Moreover, in patients with cirrhosis, a reduced intestinal motility has been described. As a matter of fact, the consequent delayed small intestinal transit could represent a risk factor for dysbiosis [77].

As a consequence of these phenomena, a decrease of Bifidobacterium and Lactobacillus has been observed in individuals with alcohol-related disorders [78]. Interestingly, both Lactobacillus spp. and Bifidobacterium spp. have been demonstrated to increase during alcohol abstinence, probably favouring alcohol withdrawal [78].

#### 3.3.2. Cigarette Smoking

Cigarette smoking is the most preventable cause of mortality for cerebro-cardiovascular diseases, cancer and chronic obstructive pulmonary disease. Although a great variability in the estimates between countries exists, worldwide prevalence of cigarettes smoking is about 25% in men and 5% in women [79].

While several studies have reported a close association between tobacco use and periodontal and respiratory dysbiosis [80,81], even suggesting that cigarette smoking could cause a shift from “good” to “bad” microbiota, to date few studies evaluated the association between gut microbiota and tobacco use. This issue is even more important if one considers the frequent association between cigarette smoking, mental health and periodontal disease [82]. To date, a decreased microbiota variability and relative abundance of Bacteroides, Prevotella, Enterobacteria and Clostridium were described among smokers. These microbiome changes may play a role in the pathogenesis of several disorders [83], potentially “reversible” after smoking cessation [84]. 

Cigarettes could be responsible for the gut microbiota alteration both in a direct and indirect way. 

Firstly, tobacco intake can determine the exposure of specific bacteria in the gut. The “microenvironment” of smokers is, in fact, characterized by a low oxygen concentration and a low pH. Facultative or obligatory anaerobic bacteria obviously predominate in this contest of “oxygen tension”, thus modifying the bacterial community [85]. Secondly, tobacco is an immunosuppressive agent able to decrease natural killer T lymphocytes and to alter macrophage and neutrophil function, increasing the susceptibilities to infection [86]. 

Finally, cigarettes can promote a chronic inflammatory status modulating the release of IL-6, INF-γ and TGF-β, generating reactive-oxygen species and increasing intestinal permeability [87].

#### 3.3.3. Cocaine Abuse

According to more recent epidemiological data, about 0.4% of the global population from 15 to 64 years is composed of cocaine users [88]. Cocaine chronic use is frequently associated with severe behavioural changes, due to craving and its direct psychoactive effects. 

Previous studies have assessed the effect of cocaine on gut microbiota, generally reporting a cocaine-induced dysregulation of the gut-barrier function leading to a microbial colonization [89]. Interestingly, the relationship between cocaine and microbiota could be defined as being “bi-directional”. Indeed, if on the one hand, an altered microbiota may enhance the sensitivity to cocaine reward and cocaine-related behavioural disturbances [90], on the other hand, cocaine administration depletes several bacteria, including Mucispirillum, Ruminococcaceae, Lachnospiracea, Pseudoflavonifractor and Butrycicoccus [91]. These bacteria are producers of SCFA, indispensable for the maintenance of mucosal and immune homeostasis. Importantly, animal studies have demonstrated that the SCFA supplementation, as well as the supplementation of other similar histone deacetylase inhibitors molecules, could attenuate cocaine-related behavioural disturbances [92]. It has been reported that cocaine administration is associated with the upregulation of pro-inflammatory cytokines, IL-18 and IL-1β in particular, contributing not only to gut-inflammation, but also to neuro-inflammation [93].

### 3.4. Molecular Mechanisms

Several molecular aspects can account for the previously reported evidence suggesting a relationship between dysbiosis, cognition and behaviour. 

From a molecular point of view, dysbiosis can exert a role in the pathogenesis and/or maintaining of behavioural disturbances, since the altered microbiota determines the consumption of substrates along with the production of metabolites, ultimately influencing brain functioning. In particular, a “healthy” gut microbiota exerts a beneficial role for the host through different mechanisms, including (1) carbohydrates and proteins digestion and fermentation; (2) production of micronutrients; (3) release of neuroactive molecules [94]. 

Concerning the carbohydrates, the intestinal microbiota transforms them into SCFAs, the anti-inflammatory properties of which have been discussed above. Moreover, it has been demonstrated that SCFAs are able to activate G protein-coupled receptors, exerting a neuroprotective function and favouring the production of neurotransmitters [95]. 

As for proteins, fermentation and bacterial putrefaction increase their metabolism, resulting in the production of both essential amino acids and toxic products (i.e., putrescine, phenol and ammonia), associated to an increased risk of depression [96]. 

Moreover, gut microbiota is responsible for the production of several water-soluble vitamins, including biotin, folate, niacin, ascorbate, riboflavin and thiamine [97]. During a condition of dysbiosis, the altered microbiota results in a reduced amount of such vitamins. Interestingly, their reduction has been associated with depression, schizophrenia and behavioural disorders [98,99].

Finally, the GIT functions are not limited to the absorption and production of metabolites, since gut microbiota can even synthetize neurotransmitters, crucial for brain functioning, mood and behaviour. More specifically, in vitro studies demonstrated that some bacteria, such as Bifidobacterium and Lactobacillus, can not only produce neurotransmitters precursors including tryptamine [100], but also active neurotransmitters, including γ-aminobutyric acid (GABA), serotonin, norepinephrine, and dopamine [101,102]. Concerning GABA, its inhibitory activity modulates several fundamental brain circuits, including the “reward” ones. Indeed, several substances of abuse, such as benzodiazepines and alcohol, directly modulate the activity of the GABA_A_ Receptors. Interestingly, it has been noted that also drugs of abuse that have non-GABA related mechanisms of action interfere with GABA_A_R genes, thus supporting the role of GABA transmission in the reward circuits [103]. Alterations of GABA signalling have also been observed in individual suffering from depression and anxiety, as well as from schizophrenia-associated cognitive impairment [103]. As far as the serotonergic and catecholaminergic (norepinephrine and dopamine) systems are considered, their impairment is widely recognized in depression, hence the most commonly prescribed drugs are the serotonin and norepinephrine uptake inhibitors [104]. Conversely, accumulating evidence suggests a relationship between the over-activation of the dopaminergic and serotonergic pathways and the cognitive-behavioural symptoms of schizophrenia [105]. 

## 4. Conclusions

As previously stated, literature often provides inconsistent data and further methodologically sound studies are needed to clarify the relationship between dysbiosis and cognitive-behavioural disturbances. Overall, however, the evidence reported in the previous paragraphs supports the pivotal role of dysbiosis in the occurrence of cognitive and behavioural disturbances. Indeed, both clinical and molecular data highlight that an altered microbiota could result in a variety of functional disturbances that affect not only the GIT, but also the whole body (including the brain tissue). Shedding light on the link between altered microbiota, cognitive performance and behaviour could provide insights regarding the pathogenesis of these burdening neuropsychiatric disorders and even suggest future therapeutic strategies.
